# Adsorption of Nucleic Acid Bases, Ribose, and Phosphate by Some Clay Minerals

**DOI:** 10.3390/life5010637

**Published:** 2015-02-27

**Authors:** Hideo Hashizume

**Affiliations:** National Institute for Materials Science, Tsukuba 305-0044, Japan; E-Mail: hashizume.hideo@nims.go.jp; Tel.: +81-29-860-4335; Fax: +81-29-860-4667

**Keywords:** adsorption, clay minerals, adenine, adenosine, 5-AMP, cytosine, uracil, ribose, phosphate

## Abstract

Besides having a large capacity for taking up organic molecules, clay minerals can catalyze a variety of organic reactions. Derived from rock weathering, clay minerals would have been abundant in the early Earth. As such, they might be expected to play a role in chemical evolution. The interactions of clay minerals with biopolymers, including RNA, have been the subject of many investigations. The behavior of RNA components at clay mineral surfaces needs to be assessed if we are to appreciate how clays might catalyze the formation of nucleosides, nucleotides and polynucleotides in the “RNA world”. The adsorption of purines, pyrimidines and nucleosides from aqueous solution to clay minerals is affected by suspension pH. With montmorillonite, adsorption is also influenced by the nature of the exchangeable cations. Here, we review the interactions of some clay minerals with RNA components.

## 1. Introduction

Because of their fine particle size, large surface area, and peculiar charge characteristics, clay minerals are capable of adsorbing and catalyzing the polymerization of various organic molecules [1]. Clay minerals have also been implicated in chemical evolution and associated with the origin of life on Earth [2,3]. Clay minerals would be easily dispersed in the ocean of the early Earth in the form of very fine particles that would adsorb organic molecules dissolved in the ocean. Thus, organic molecules would be deposited and concentrated on the ocean floor. Organic molecules might be able react with each other via the catalytic activity of clay minerals. Organic molecules might be formed as bioorganic molecules or biopolymers. It is therefore hypothesized that clay minerals could have played an important role in the origin of life. 

Clay minerals are defined as very fine particles (less than 2 μm in diameter) [4]. Clay minerals are generally formed by weathering of rock-forming minerals. Fragmented minerals are also classified as clay minerals. The chemical compositions of clay minerals are mainly hydrosilicate and metal hydroxide. Configurations of clay minerals are mainly as a sheet, tube, or spherule. Clay minerals used for studies of the origin of life are generally a form of sheet silicate, such as smectite and kaoline minerals. 

Indeed, clay minerals could function as a primitive form of genetic material, capable of replicating information stored in the form of structural, layer charge, and layer stacking faults [2,5,6]. Bioorganic molecules, polymerized into biopolymers in the interlayers of clay minerals, might replicate with the aid of the mineral host over time. This hypothesis is unique, but its validity is difficult to demonstrate experimentally [6]. 

Polynucleotides formed in such a manner could transform into a proto-RNA, and function as both a storehouse of genetic information and an enzyme (catalyst) in the primitive “RNA world” [7,8]. Here again, no experimental evidence exists to indicate that clay minerals can catalyze the formation of nucleotides from nucleic acid bases, ribose, and phosphate, let alone that of polynucleotides. However, oligomers of RNA monomers, “activated” by an imidazole or a methyl group attached to the phosphate group, can form in the presence of montmorillonite. Under certain conditions, RNA with up to 50 monomers can be obtained by these means [9,10,11].

The interaction of clay minerals with biomolecules has attracted a great deal of attention. In early studies, Lailach *et al*. [12,13] determined the adsorption of purine and pyrimidine bases by montmorillonite as a function of solution pH. Adsorption of adenine and cytosine decreased with increasing pH, becoming negligibly small at pH ≈ 8. More recently, Benetoli *et al*. [14] reported that more adenine, cytosine, uracil, and thymine were adsorbed by montmorillonite more at pH 2 than at pH 7.2. 

Here, we summarize adsorption of RNA components with some clay minerals, including smectite, kaoline minerals, allophane, and layered double hydroxide.

## 2. Smectite

**Figure 1:**
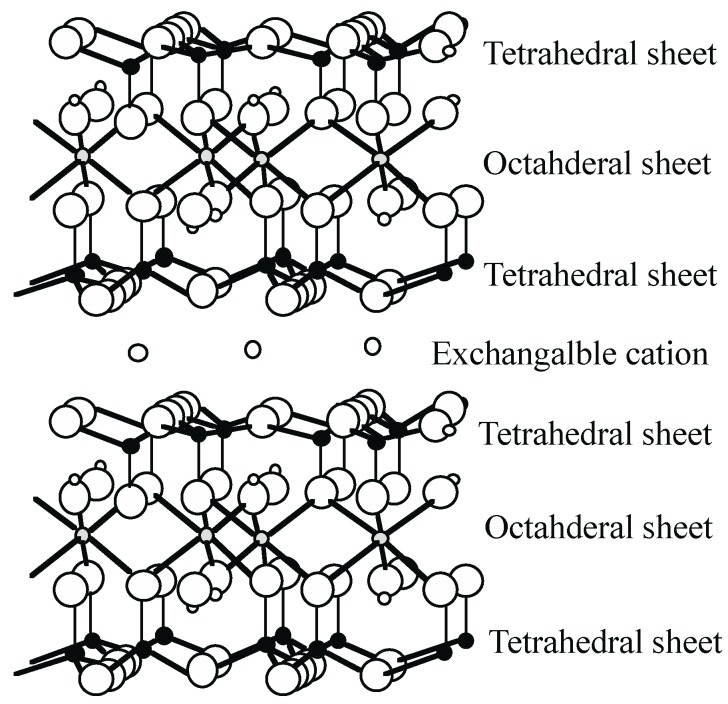
The layer structure of smectite group.

Smectite is a group including montmorillonite, beidellite, nontronite, saponite, hectorite, sauconite, and stevensite. The general structure of smectite is shown in Figure 1. Montmorillonite, beidellite, and nontronite are called dioctahedral smectite. The octahedral site is normally occupied by trivalent cations (e.g., Al^3+^, Fe^3+^, Mn^3+^). Saponite, hectorite, sauconite, and stevensite are called trioctahedral smectite. The octahedral site usually has divalent cations (e.g., Mg^2+^, Fe^2+^, Mn^2+^, Ni^2^^+^). Si^4+^ and Al^3+^ are mainly present in the tetrahedral site. One of the typical characteristics of the smectite group is the presence of an exchangeable cation (e.g., Na^+^, K^+^, Ca^2+^) between layers and smectite swells by intercalating water molecules between layers. Smectite can easily disperse in water and the interlayer cations can exchange with ionic organic molecules. Montmorillonite is frequently used in studies of the origin of life. The planar surface usually has a negative charge. The edge surface of montmorillonite has an isoelectric point (pH approximately 7). At pH < 7, the edge surface shows a positive charge and at pH > 7, it has a negative charge [15].

Lailach *et al*. [12,13] showed adsorption of adenine, cytosine, and other related organic materials at various pH values by cation-exchanged montmorillonites, of which interlayer cations were Li^+^, Na^+^, Mg^2+^, and Ca^2+^. Almost all adsorbates were adsorbed by montmorillonite at a low pH. Adsorption suddenly decreased around pH 5 and at pH 8, montmorillonite did not adsorb these organic molecules. No major difference in adsorption by the interlayer cations was observed.

Perezgasga *et al*. [16] investigated the blocking effect of hexadecyltrimethyl ammonium cation (HDTMA) on adsorption of adenine and uracil by Na^+^-montmorillonite at pH 2, 6, and 10. Without HDTMA, 98% of adenine was adsorbed at pH 2, but at pH 6 and 10, adenine was not adsorbed. Conversely, 37% of the adenine was adsorbed at pH 2 with HDTMA. HDTMA blocked the intercalation of adenine. For uracil, 30% was adsorbed by montmorillonite. The effect on HDTMA decreased uracil adsorption from 30% to 6%. The extent of adenine adsorption decreased from 98% to 48% due to the addition of either HDTMA or phosphate. At pH 2, the adsorption achieved an equilibrium concentration for only 15 min. 

Winter and Zubay [17] studied adsorption of adenine or uracil in a buffered solution and a model seawater. The buffer consisted of disodium salt, piperazine-N, N-bis-2-ethanesulfonic acid, NaCl, and MgCl_2_ at pH 6.7. The seawater model comprised sodium, magnesium and potassium colloid, and either sulfate or carbonate at pH 8.6. Adenine was well adsorbed in the buffer and seawater model at either a neutral or basic pH. The maximum adsorption of adenine was approximately 0.18 mmol·g^−1^ of about 0.2 mmol·dm^−3^ of the equilibrium concentration of solute, in buffering condition, and 0.08 mmol·g^−1^ of approximately 1.8 mmol·dm^−3^ in the seawater model. Approximately 0.27 mmol·g^−1^ of uracil was adsorbed at about 13 mmol·dm^−3^ by montmorillonite. Adenine adsorption was increased compared with uracil in the seawater model.

Hashizume *et al*. [18] previously determined the isotherms for the adsorption of adenine, cytosine, uracil, ribose, and phosphate by Mg^2+^-exchanged montmorillonite at pH 7–8. In their experimental condition, all isotherms were approximately linear lines. Under standard conditions, adsorption decreases in the following order: adenine > cytosine > uracil. The extent of adenine adsorption was 0.08 mmol·g^−1^ at 4 mmol·dm^−3^; cytosine adsorption was 0.15 mmol·g^−1^ at 15 mmol·dm^−3^ and uracil adsorption was 0.03 mmol·g^−1^ at 18 mmol·dm^−3^. Winter and Zubay [17] observed that adsorption of adenine and uracil was higher than values reported by Hashizume *et al*. [18]; however, their respective experimental conditions were different.

Mechanisms of nucleic acid base adsorption were described by Hashizme *et al*. [18] and Perezgasga *et al*. [16]. Their observations may be explained in terms of differences in acid dissociation constant (pKa), solubility, and molecular weight (size) of the various compounds (Table 1). Nucleic acid bases are apparently intercalated by H-bonding with water molecules. As such, adsorption would decrease with an increase in basicity (pKa value), in line with experimental observations. Generally, adsorption of a solute decreases as its solubility (in water) increases, and increases with molecular weight. Cytosine is more soluble in water than uracil, while the molecular weight of cytosine is nearly equivalent to uracil. Nevertheless, more cytosine than uracil is adsorbed, suggesting that basicity is the determining factor. In the case of adenine, however, both molecular weight and basicity contribute to its relatively high adsorption.

**Table 1:** Dissociation constant, solubility, and molecular weight of adenine, cytosine, uracil, adenosine, ribose, and 5'-AMP. (T) in solubility is temperature of measurement.

		Adenine	Cytosine	Uracil	Adenosine	Ribose	5'-AMP
Dissociation constant	pKa_1_	4.15 *	4.58 ^+^	9.48 ^+^	3.5 *		3.80 ^#^
	pKa_2_	9.8 *	12.15 ^+^		12.5 *		6.19 ^#^
	pKa_3_						13.06 ^#^
Solubility (T) (g/100g)		0.12 (25 °C) ^†^	0.743 (?)·	0.36 (25 °C) ^†^	0.7 (?) ^§^	10 (?) ^††^	97 (25 °C) ^^^
Molecular weight		135	111	112	267	150	347

Dissociation constant; * [17]. ^+^ [19]. ^#^ [20]. Solubility; ^†^ [21,22]. ^§^ [23]. ^††^ [24]. ^ [25]. ? is not shown.

**Figure 2:**
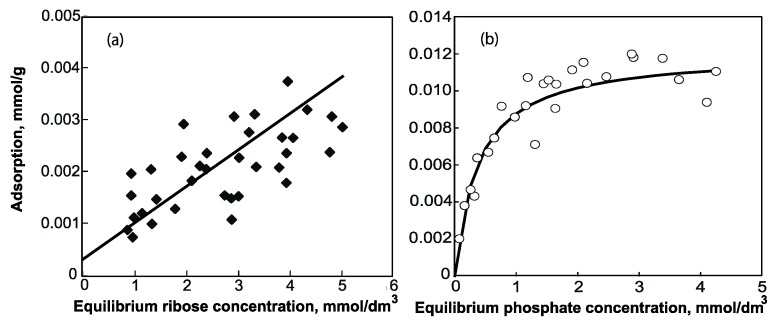
Isotherms for the adsorption of ribose (**a**) and phosphate (**b**).

The isotherm for the adsorption of ribose by Mg^2+^-montmorillonite is shown in Figure 2a. The points are scattered and very little is adsorbed because ribose is highly basic (pKa = 12.2). Furthermore, ribose would be negatively charged at the experimental pH (≈ 8), and hence be repelled from the (basal) silicate surface. The isotherm for the adsorption of phosphate (Figure 2b) is of the L-type [26], reaching a plateau when the solute concentration exceeds 1.2 mmol·dm^−3^. At an acidic pH, phosphate can adsorb by electrostatic attraction to the edge surface of montmorillonite particles, which is then positively charged because the isoelectric point of the edge surface of montmorillonite is around pH 6.5 [27]. However, at pH ≈ 8, both the basal and edge surfaces of montmorillonite are negatively charged. Under these conditions, phosphate can still adsorb by ligand exchange with hydroxyl groups attached to “under-coordinated” aluminum ions at particle edges (Figure 1). 

For pH dependence on nucleoside adsorption by montmorillonite, Li^+^-, Na^+^-, Mg^2+^-, and Ca^2+^-montmorillonite adsorbed adenosine well at less than pH 4. Over pH 4, adsorption by Li^+^- and Na^+^-montmorillonite decreased gradually until pH 8, but adsorption by Mg^2+^- and Ca^2+^-montmorillonite decreased very quickly and at pH around 6, adenosine was not adsorbed at all. Adsorption of guanidine decreases from pH 3. Adsorption by Li^+^- and Na^+^-montmorillonite decreased gradually and around pH 7, adsorption did not occur. Mg^2+^- and Ca^2+^-montmorillonite did not gradually adsorb guanidine around pH 4. Cytidine adsorption by Li^+^- and Na^+^-montmorillonite was similar to adenosine but adsorption by Mg^2+^- and Ca^2+^-montmorillonite was lower compared with adenosine. Montmorillonite with a divalent cation achieved lower adsorption of adenosine, guanidine, and cytidine compared with a monovalent cation [12,13].

Winter and Zubay [17] investigated adenosine adsorption by montmorillonite with use of a buffer. They observed that adenosine adsorption was lower compared with adenine at pH 2 in the buffering condition. Thus, the buffer interfered with adenosine adsorption. 

Nucleotide adsorption is also affected by pH. Lawless *et al*. [28] evaluated adsorption of 5'-, 3'- and 2'-adenosine monophosphate (AMP) by montmorillonite with various divalent cations in interlayers. The extent of adsorption of 5'-AMP by Zn^2+^-montmorillonite displayed two maximal peaks at pH 3 and 7 with increasing pH. Adsorption of 3'- and 2'-AMP also displayed two small peaks with increasing pH, but these were relatively small. Adsorption of 5'-CMP showed a similar tendency as 5'-AMP. 5'-AMP was more adsorbed compared with 5'-CMP. For Cu^2+^ as the exchangeable cation, the adsorptive profile of 5'-AMP was similar as Zn^2+^-montmorillonite. For other interlayer cations (Na^+^, Mn^2+^, Fe^3+^, Co^2+^, and Ni^2+^), adsorption of 5'-AMP decreased with increasing pH; no peaks were observed. The difference between Zn^2+^ or Cu^2+^ montmorillonites and other montmorillonites might be associated with altered coordination. Cu^2+^ and Zn^2+^ display a four-fold coordination, whereas Mn^2+^, Fe^2+^, Co^2+^, and Ni^2+^ have a six- fold coordination. Banin *et al*. [20] investigated adsorption of 5'-AMP by montmorillonite, in which the interlayer cation changed from 100% Ca^2+^ and 0% Fe^2+^ to 0% Ca^2+^ and 100% Fe^2+^ at different pH values. Fe^2+^-rich montmorillonite adsorbed more 5'-AMP compared with Ca^2+^-rich montmorillonite. Adsorption decreased with increasing pH regardless of the interlayer cations.

Adsorption of AMP, ADP, and ATP by montmorillonite at pH 2 was investigated at different times by Perezgasga *et al*. [16]. It took approximately 15 min to achieve an adsorptive equilibrium. Adsorption was lower than that of adenine. In addition to HDTMA or phosphate in the solution, adsorption was lower than without addition. Winter and Zubay [17] also investigated adsorption of 5'-AMP, ADP, and ATP in the buffered solution at pH 6.7. Adsorption decreased in the following order: ATP > ADP > AMP. Feuillie *et al*. [29] studied the adsorption of AMP, GMP, CMP, UMP, and dGMP by montmorillonite and nontronite at pH 6.5. Compared with isotherms for adsorption by montmorillonite and nontronite, isotherms by nontronite were higher compared with montmorillonite for the five nucleotides. At pH 6.5, nucleotides would be adsorbed on the edge surface because of a positive charge. Conversely, the planar surface always has a negative charge. Thus, the nucleotide could not be adsorbed on the planar surface at pH 6.5. The authors expected the edge surface of nontronite to be different compared with montmorillonite.

Adsorption of polynucleotides by montmorillonite with a different interlayer cation (Na^+^, Ca^2+^, or Mg^2+^) was carried out by Franchi *et al*. [30]. Na^+^-montmorillonite did not adsorb polyadenylic acid (polyA), polyuridylic acid (polyU), polydeoxyadenylic acid (polydA), polydeoxythymidylic acid (polydT) and chromosomal DNA (DNAchr). However, Ca^2+^- or Mg^2+^-montmorillonite almost completely adsorbed them from about 1 mmol·dm^−3^. The authors explained that the divalent cations played a bridging role between the montmorillonite surface and the polynucleotide.

Montmorillonite can adsorb nucleic acid bases, nucleosides, nucleotides, and polynucleotides. In the early Earth, high-energy radiation and UV light would come from space. Biomolecules might be destroyed by such radiations. Biondi *et al*. [31] and Agnilar-Orando and Negron-Mendoza [32] studied the decomposition of adenosine and RNA by irradiation of ^60^Co γ-ray or 254 nm UV ray when they were adsorbed by montmorillonite or they were by themselves. When both adenosine and RNA was adsorbed by montmorillonite, they remained at a high concentration compared with the biomolecule by themselves, indicated a protective effect of the montmorillonite.

Theoretical or computer simulation studies on adsorption of nucleic acid bases, nucleotides, and polynucleotides were also reported. For example, Mignon *et al*. [33] simulated the adsorptive energies and distance between nucleic acid bases and a Na^+^- montmorillonite surface. The morphology of adsorption between nucleic acid bases and montmorillonite affected the adsorptive energy. The energies of face-to-face nucleic acid bases and montmorillonite were generally higher than those of cations-π bonding. Mathew and Luthey-Schulten [34] computed the relation of a nucleotide to di-nucleotide within and without an interlayer of Ca^2+^-montmorillonite. They indicated that the reaction from monomer to dimer was made better in the interlayer than out of the interlayer. In addition, a 3'–5' reaction of ribose was superior to 2'–5' reaction within the interlayer. Swadlng *et al*. [35] studied the interaction between montmorillonite and RNA. When RNA was adsorbed on a montmorillonite surface, the RNA conformation changed and shrank because of the surface charge. Montmorillonite might affect the formation of polynucleotides or RNA synthesis.

Joshi *et al.* [36] investigated the catalytic activity of montmorillonites in three regions (Whyoming, Otay, and Chambers). These montmorillonites are different from the adsorptive activity of adenine-5'-phophorimidazolide (impA), impC, and impU. Three montmorillonites were different for preservation of impA, which has negatively charged nucleotide derivatives. There are many published articles that describe polymerization catalyzed by montmorillonite, especially the work of Ferris and colleagues [37,38].

## 3. Kaoline Minerals 

Kaoline minerals are kaolinite, dickite, and nacrite. Kaoline minerals are composed of Al, Si, O, and OH. The chemical formula is Al_2_Si_3_O_5_(OH)_4_. The structure overlaps with an octahedral (O) and a tetrahedral sheet (T), alternatively. One layer is T-O and there are not any exchangeable ions. However, cations can intercalate into interlayers of kaoline minerals following treatment with alcohols or surfactants. The point of zero charge (PZC) of kaolinite is pH 4 [39]. Kaolinite has a positive charge under pH 4. Conversely, at a pH over 4, kaolinite has a negative charge. 

Benetoli *et al*. [14] studied the adsorption of adenine by kaolinite and montmorillonite. They showed Langmuir and Freundlich parameters of isotherm for adsorption. Kaolinite adsorbed adenine at pH 2. The maximum extent of adsorption by kaolinite was much lower compared with montmorillonite. The maximum adsorption by kaolinite was 0.407 μg·mg^−1^, while that of montmorillonite was 21.7 μg·mg^−1^. Evaluation of adsorption of 5'-AMP, ADP, and ATP by kaolinite at neutral pH was performed by Graf and Lagaly [40]. Adsorption increased in the following order: ATP < ADP << AMP. Adsorption of AMP by kaolinite was nearly equivalent to that of Ca^2+^-montmorillonite and higher than that of beidellite. However, beidellite adsorbed ADP and ATP better than kaolinite. 

Franchi *et al*. [30] investigated adsorption of polyA, polyU, polydA, polydT and DNAchr by kaolinite, and modified kaolinite at pH 5 to 5.5, which Ca^2+^ and Mg^2+^ were intercalated in the interlayers. Kaolinite itself hardly adsorbed those polymers at a low concentration. At high concentrations, kaolinite adsorbed about 80% of the polymers except DNAchr. Conversely, kaolinite with a divalent cation adsorbed almost all of the polymers over 1 mmol·dm^−3^. Thus, Mg^2+^ and Ca^2+^ cations could help connect kaolinite and polymers, similar to the interaction between montmorillonite and polymers. 

Computer simulations of adsorption of thymine and uracil by kaolinite and dickite were carried out by Robinson *et al*. [41] and Michalkova *et al*. [42], respectively. The interaction energy between uracil or thymine and kaolinite in sodium solution was calculated by Robinson *et al*. [41]. The energy of uracil was lower compared with thymine. The difference between the energies of uracil and thymine was very small. The interaction energy between dickite and uracil was also smaller than that of thymine. In kaoline minerals, the interaction energy can be investigated between nucleic acid bases and a tetrahedral surface or an octahedral surface. The interaction energy between the octahedral surface and uracil or thymine was lower compared with a tetrahedral surface. The octahedral site has a vacancy for the charge valance. The vacancy might affect the different interaction energy between the octahedral and tetrahedral surfaces.

## 4. Allophane and Other Silicates

**Figure 3:**
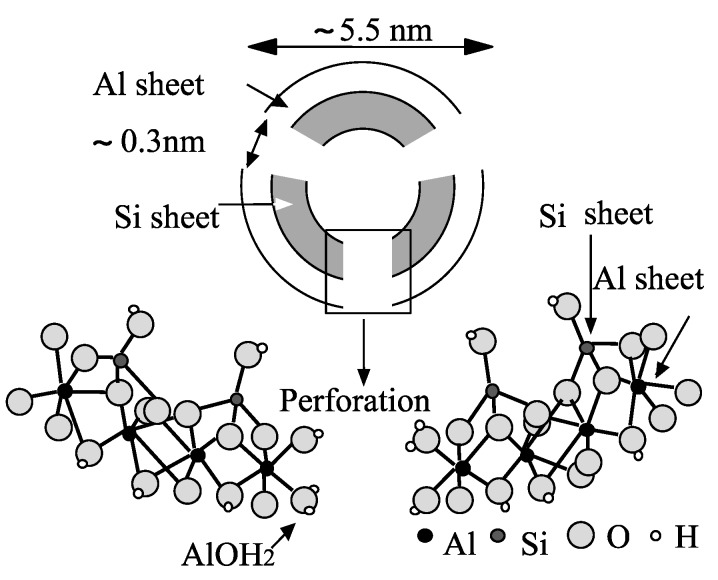
Diagram showing the hollow spherule structure of an allophane particle (“nanoball”), and composition of the (perforated) spherule wall. See Figure 1 in [44].

Allophane is a nanosized hydrated aluminosilicate with short-range order and an Al/Si ratio of 1 ~ 2 found in many soils derived from volcanic ash and weathered pumice. The unit particle of allophane consists of a hollow spherule with an outer diameter of 4–5.5 nm. The 0.7–1.0 nm thick spherule wall is composed of an outer gibbsitic sheet, to which O_3_SiOH groups are attached on the inside. Defects in the wall structure give rise to ~0.3 nm wide perforations (Figure 3). Unlike montmorillonite, allophane has neither a permanent negative charge nor exchangeable cations. Rather, the charge characteristics of allophane vary with pH, since the (OH)Al(OH_2_) groups exposed at wall perforations can either acquire or lose protons, depending on suspension pH. The point of zero charge of the allophane sample, used in this instance, is close to 6 [43]. 

**Figure 4:**
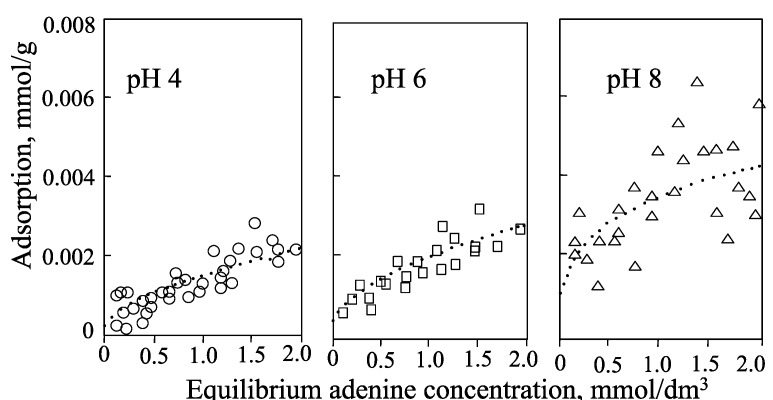
Isotherms for the adsorption of adenine by allophane at pH 4, 6, and 8.

**Figure 5:**
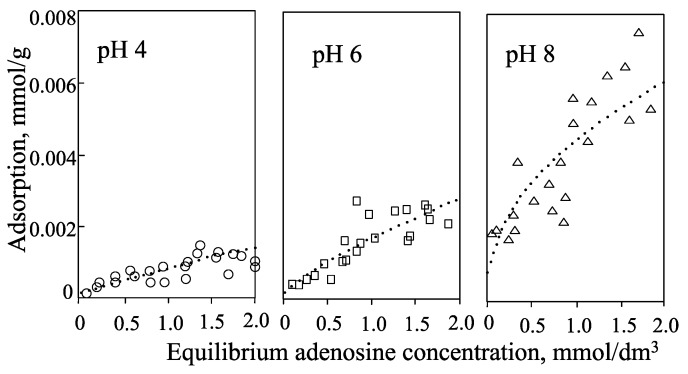
Isotherms for adsorption of adenosine by allophane at pH 4, 6, and 8.

**Figure 6:**
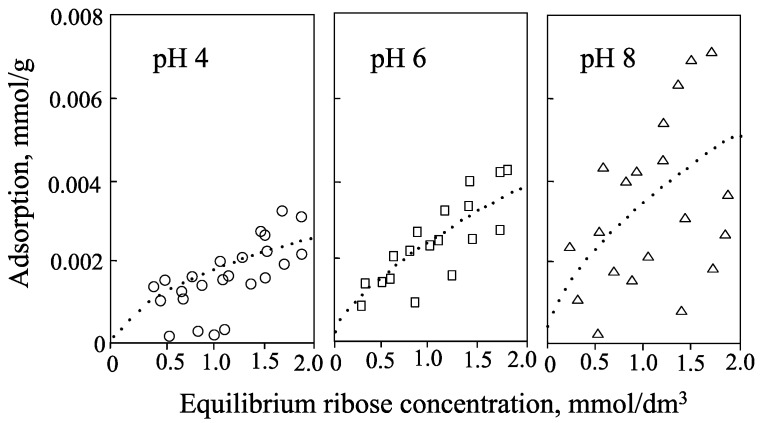
Isotherms for adsorption of ribose by allophane at pH 4, 6, and 8.

**Figure 7:**
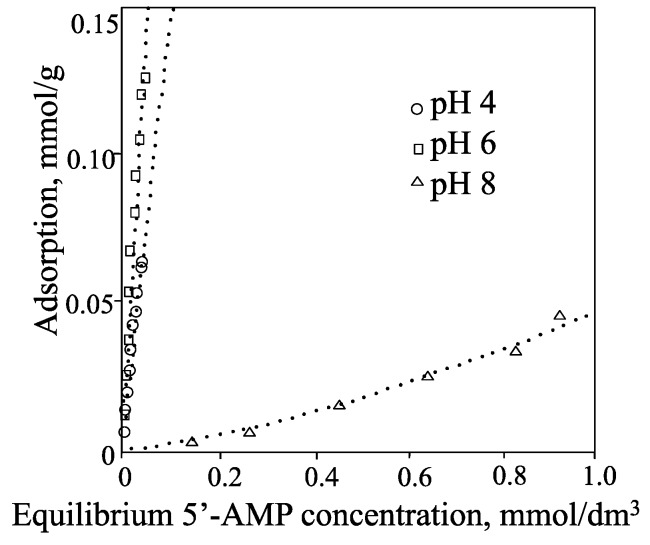
Isotherms for the adsorption of 5'-AMP by allophane at pH 4, 6, and 8.

Hashizume and Theng [44] have determined the isotherms for the adsorption of adenine, adenosine, ribose, and 5'-AMP by allophane at pH 4, 6, and 8 (Figure 4, Figure 5, Figure 6 and Figure 7). Little adenine is adsorbed at all three pH values. As for adenine, adsorption of adenosine increases in the following order: pH 4 < pH 6 < pH 8 (Figure 5). A similar trend was observed with ribose (Figure 6). The adsorption of adenosine would be affected by the adenine component of adenosine. The ribose component of adenosine did not affect adenosine adsorption. 

The isotherms for the adsorption of a 5'-AMP at pH 4, 6, and 8 are shown in Figure 7. At pH 4 and 6, the amount adsorbed at low solute concentrations (<0.05 mmol·dm^−3^) was at least two orders of magnitude greater than that for adenosine. Even at pH 8, when the allophane surface is negatively charged, appreciable adsorption of 5'-AMP was observed (Table 2). According to Theng *et al*. [45] and Rajan [46], the dramatic increase in adsorption may be ascribed to ligand exchange between the phosphate group of 5'-AMP and the hydroxyl of (OH)Al(OH_2_) groups on the surface of allophane spherules (Figure 3), forming monodentate and bidentate surface complexes [44]. Allophane was a superior clay mineral of nucleotide adsorption compared with montmorillonite. In a recent study of the interaction of DNA with allophane, Matsuura *et al*. [47] showed that allophane was adsorbed on single-stranded DNA (ss-DNA). The authors also mentioned that the phosphate group of ss-DNA strongly associated with Al-OH group of allophane. 

**Table 2:** Charge characteristics of adenine, adenosine, 5'-AMP, and allophane at pH 4, 6 and 8 [20,44].

	pH 4	pH 6	pH 8
adenine	++	(+) N	N (+)
adenosine	++	(+) N	N
5'-AMP	+−	−	−−
allophane	++	N	−−

+: positive; −: negative; N: neutral; N(+): weakly positive; ++: strongly positive; −−: strongly negative

Graf and Lagaly [40] investigated adsorption of AMP, ADP, and ATP by illite, quartz and silt-quartz. Illite is included in a mica group. The crystal structure is similar to smectite (Figure 1). The ideal chemical composition is K_0.75_(Al_1.75_(Mg,Fe^2+^)_0.25_)(Si_2.5_Al_0.5_)O_10_(OH)_2_. The interlayer cation does not basically exchange to other cations. The intercalation into interlayers does not occur. Quartz is composed of SiO_2_. Silt of quartz-silt means a particles size. The silt is 2–20 μm in diameter, according to The International Soad Method of the International Society of Soil Science [4]. The authors mentioned that illite hardly adsorbed AMP, ADP, or ATP. In the case of quartz, they compared adsorption by quartz to that by quartz-silt. Quartz-silt did not contain AMP, ADP and ATP after a removing treatment.

## 5. Layered Double Hydroxide (LDH) 

**Figure 8:**
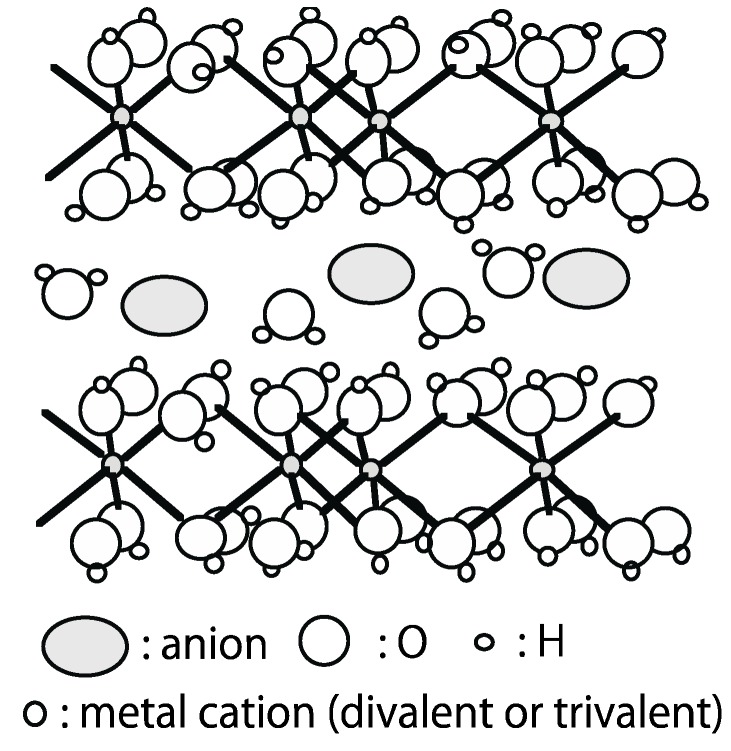
Schematic figure of Layered Double Hydroxide (LDH) structure.

LDH is a clay mineral that can exchange anions. The crystal structure is shown in Figure 8. Natural LDHs have divalent and trivalent cations and the interlayer is occupied by anions (Table 3). 

**Table 3:** Natural LDHs with various divalent and trivalent cations and interlayer anions [53].

Mineral name		Cation		Anion
Rhombohedral	Hexagonal	M^2+^	M^3+^	
Hydrotalcite	Maasseite	Mg	Al	CO_3_^2−^
Motukoreaite		Mg	Al	SO_4_^2−^, CO_3_^2−^
Stichtite	Barbertonite	Mg	Cr	CO_3_^2−^
Pyroaurite	Sjögrenite	Mg	Fe	CO_3_^2−^
Iowaite		Mg	Fe	Cl^−^
Chlormagaluminite		Mg	Fe	Cl^−^, CO_3_^2−^
	Hydrocalmite	Ca	Al	OH^−^
Green Rust 1		Fe	Fe	CO_3_^2−^
Berthierine		Fe	Fe	SiO_4_^4−^
Takovite		Ni	Al	CO_3_^2−^
Reevesite		Ni	Fe	CO_3_^2−^
Honessite		Ni	Fe	SO_4_^2−^
Eardlyite		Zn, Ni	Al	CO_3_^2−^
Meixnerite		Mg	Al	OH^−^

LDH has often been investigated in association with sugars, polynucleotides, RNA. Aizawa *et al*. [48] studied the synthesis of LDH in a ribose solution. LDH was composed of Mg^2+^ and Al^3+^ cations containing ribose between layers, while Zn^2+^ and Al^3+^-LDH could not be formed in ribose solution. Swadling *et al*. [49] and Swadling *et al*. [50] calculated the stability of RNA, DNA, and PNA in the interlayer of LDH and showed that DNA was the most stable. The authors also simulated the bonding between RNA and the surface of LDH composed of Mg^2+^ and Al^3+^. They showed that the phosphate of RNA connected with the LDH surface. The interlayer of LDH was used to synthesize sugar-phosphate in other reactions [51,52].

## 6. Conclusions 

We have shown that some clay minerals can adsorb RNA components. Although the extent of their adsorption is rather limited, the results provide valuable information about the mechanisms underlying the interaction between clay minerals and simple bioorganic compounds. Organic molecules, synthesized abiotically in the ocean [54,55,56,57], could be immobilized and concentrated on clay mineral surfaces. The resultant organic-rich mineral particles would sink and accumulate on the ocean floor. The ability of clay minerals to take up and concentrate key components of RNA has been experimentally demonstrated. A more pertinent point is the large capacity of allophane for binding and retaining phosphate, as phosphorus is not major element making up the chemical composition of the Earth’s crust. Organic molecules, including polymer adsorbed on clay minerals, might be protected from radiation from UV rays and cosmic rays [31,32]. 

Some clay minerals are useful for nucleotide polymerization [58]. It is also difficult to form nucleoside and nucleotide in the prebiotic condition. Even if clay minerals such as montmorillonite and kaolinite, exist with nucleic acid bases, ribose and phosphate, nucleosides and nucleotides can not formed, easily. There are several hundred kinds of clay minerals and other minerals in total. Some clay minerals in them might have a function for the formation of nucleosides and nucleotides as a catalyst. As the basic investigation, it is important to study adsorption of RNA components using various clay minerals.
